# Defense Mechanism Functioning in Patients With Breast Cancer: Using the Defense Mechanism Rating Scale

**DOI:** 10.3389/fpsyg.2021.666373

**Published:** 2021-08-11

**Authors:** Marwa Saab, Matias Hartmann, Xue Han

**Affiliations:** School of Psychology, Northeast Normal University, Changchun, China

**Keywords:** breast cancer, defense mechanism, adaptive mechanism, chronic disease, cancer stage, Lebanon

## Abstract

**Background:** Breast cancer (BC) is one of the highest incidence rates in Lebanon. Previous studies had focused scarcely on the unconscious protective shield of patients with BC or BC survivors against cancer, while only some studies had focused on the relationship between defense mechanisms (DMs) and high adaptation with the disease process and progress. Therefore, this study aimed to investigate the reaction of inpatients with BC toward the disease by measuring DMs in the Lebanon context.

**Methods:** Seventy inpatients with BC were recruited randomly from six hospitals. Their DMs were measured using the Defense Mechanism Rating Scale. Moreover, the Relationship Anecdotes Paradigm (RAP) was used with three recent life vignettes of initial diagnosis, family, and daily life. The data were analyzed using repeated measure ANOVA, Wilcoxon rank-sum test, and Spearman's rank.

**Results:** Patients with stage 2 BC used more high-adaptive defense levels than patients with stage 4 BC who used more minor image distorting defense levels. Moreover, patients with stage 2 BC used more self-observation and undoing, while patients with stage 4 BC used more devaluation.

**Conclusion:** The severity of BC, the age of carriers, and the social status may lead to higher use of DMs, at the level of the individual defense and the hierarchal or the tripartite levels.

## Introduction

Cancer is the second leading cause of death worldwide [World Health Organization (WHO), 2020a]. In Lebanon, cancer was merely responsible for 16% of death in 2016, and breast cancer (BC) was the one with the highest incidence cancer rates. BC was merely responsible for 19% of the total cancer cases in 2016, with an increase to 24% between 2007 and 2016 [Lebanese Ministry of Public Health (MOPH), 2020]. There were 17,294 new cancer cases in Lebanon in 2018, of which BC accounted for 18.6% of these new cases and had the highest new incidence rate in women with 37.9% [World Health Organization (WHO), 2020b].

Although previous studies showed that patients with BC have high anxiety and depression levels and the anxiety was associated with physical burdens (Park et al., 2018), women patients with BC were shown to have higher resilience and mood repair levels than women with non-BC (Guil et al., 2020). However, more pain, fatigue, and decreased body image were associated with decreased quality of life of women with BC (McClelland et al., 2015). Studies also found that patients with BC have hope and trust from their health professionals, who were the primary source of information (Lewis et al., 2015). However, a developed BC or metastasis stage limited social support due to the anticipation of patients with BC of an adverse reaction from their surroundings (Ginter, 2020). Vilhauer (2011) showed that women with metastatic BC who were in an online mixed-diagnosis support group felt less support than the primary stages of BC. They could not relate themselves to individuals of primary stages of BC, and they did not want to share their concerns in order to not create fear in primary survivors. Therefore, women with metastatic BC preferred the metastatic support group. However, the study by Lewis et al. (2015) showed that support groups had both positive and negative experiences among women with metastatic BC. Sharing the same experience with another person of the same concerns was positive; however, some women with BC did not want to be continuously reminded of their situation. Even though women with BC reflected the need for a support group, they preferred discussing strategies to manage cancer instead of symptoms.

Studies about BC had little focus on how patients with BC react to the disease in their daily life unconsciously. Previous studies focused more on coping mechanisms that patients used consciously, for example, age, absence of radiation therapy, time since diagnosis, and distress, which were found to be predictors of self-efficacy for coping with BC (Mosher et al., 2010). Other studies found an association between self-efficacy and seeking medical information (Collie et al., 2005), or between posttraumatic growth and depression prevention in BC survivors (BCS) (Kuswanto et al., 2020). In contrast, fear of reoccurrence in BCS was associated with depression, anxiety, and stress (Kuswanto et al., 2020). However, Martino et al. (2020b) found a decrease in depression and anxiety levels and an increase in perceived quality of life after the introduction of an aromatase inhibitor therapy for BCS. Yu and Sherman (2015) found that BCS use communication avoidance to cope with BC; however, they perceived that their partners use higher communication avoidance than themselves. Moreover, communication avoidance in BCS and their partners negatively correlated with engagement coping and positively correlated with depression, anxiety, stress, and self-distraction. In contrast, Weihs et al. (2008) found that close relationships and emotional processing in the first year of BC could protect against disease progression. However, these studies did not measure how patients with BC or BCS react to BC unconsciously, i.e., what defense mechanisms (DMs) they use to cope with BC.

Freud (1961) was one of the first psychologists who pointed out that humans can create a protective mental shield. According to Freud (1961), this protective shield is the protection of ego from incompatible ideas is by using the DM of transposition of the affect into psychic or somatic symptoms. He defined DM as a shield of protection from external stimuli to reduce its impact (Freud, 1962). The definition of DM is a shield that an individual unconsciously creates against the feelings of fear, guilt, and shame aligned with several other psychologists (Freud, 1972; Perry and Copper, 1989; Perry, 1990; Cramer, 1991, 2006). Moreover, according to Cramer (1991, 2006), individuals under stress tend to use lower DMs than the individuals who are not undergoing stress.

Some studies on DMs had focused on the relationship between the DM and treatment from somatic diseases (Perry and Copper, 1989; Perry, 1990; Vaillant, 1993). Martino et al. (2020a) showed that patients with type 2 diabetes mellitus tended to use higher introjections, repression, denial, and reaction formation compared with non-diabetic individuals. Additionally, males with type 2 diabetes mellitus used higher isolation, rationalization, and intellectualization compared with non-diabetic males. Perry et al. (2015) found out that women with BC use less adaptive, less neurotic, and higher immature defenses and less overall defense functioning (ODF) than women with non-BC. Moreover, physical function, emotional function, and marital status were correlated with high-adaptive defenses and ODF, in both participants with BC and non-BC. However, this study showed no significant difference between newly diagnosed and late cancer stages of patients with BC (Perry et al., 2015). Beresford et al. (2006) also did not find a significant difference in DMs between cancer stages; however, they found that mature defenses can predict survival probability in patients with cancer. Another study by Porcerelli et al. (2017) involving 49 outpatients with BC receiving radiation with or without chemotherapy investigated the relationship between utilization of medical services and projection, denial, and identification of DMs. Projection was positively correlated with the number of extra outpatient visits and the number of trips to the emergency department. In contrast, denial predicted the visits of fewer outpatients. Psychological distress was also correlated with the number of trips to the emergency department and hospitalization. Talepasand and Mahfar (2018) found a relationship between DM and quality of life. According to their empirical results, a higher prevalence of displacement, regression, reaction formation, and projection of DM in patients with BC was associated with lower cognitive and role aspects of quality of life. Renzi et al. (2017) had found that patients with BC with overprotective parents used more fantasy and withdrawal as DM, while patients with BC with less protective parents used more repression. They argued that repression might be beneficial for a patient to overcome BC.

Studies on BC in societies like Lebanon are scarce. Similarly, studies that focus on patients with BC, BCS, or metastatic DM function are scarce. Therefore, considering the high BC rate in Lebanon, the objective of this study was to investigate the unconscious protective shield of patients with BC during their disease process and progress. Thus, the difference in DMs displayed between patients with stages 2 and 4 BC was measured in the Lebanese social context. The aim of this study was also to shed some light on psychological counseling or therapy in the societies such as the Lebanese.

## Materials and Methods

### Participants

Seventy inpatient women with BC (age range: 31–81 years and mean age = 52.88 years) were randomly recruited from six hospitals (i.e., four private and two public hospitals) in Lebanon. The participants were recruited through random sampling from hospitals according to the following criteria: (1) any patient who is diagnosed by a physician as having BC, (2) undergoing therapies, (3) above 18 years old, (4) having Lebanese citizenship, and (5) speaking Arabic fluently (the official language of Lebanon). Before initiating data collection, the files of patients in hospitals were checked to obtain their medical information regarding stages. We found that all of the patients were in either stage 2 (*n* = 31) or stage 4 (*n* = 39), based on the diagnosis by their doctors. Moreover, during data collection, each patient was asked about her current stage and all of them knew their current stage.

The demographic information of participants was presented in Table 1. Furthermore, the majority of participants (75.7%) did not perform a reconstruction of the breasts after the surgery, while only 7.1% did, and 17.1% were still under chemotherapy or prior to surgery; thus, the decision for reconstruction is postponed. Moreover, more than a quarter (27.1%) of participants had no monthly income; they rely on other family members or help from the surroundings. In addition, 41.5% of the sample had no social insurance; they rely on the Ministry of Public Health support in their medication and treatment compromising 41.4%, with 0.01% self-covering all expenses.

**Table 1:** Demographic information of patients with stages 2 and 4 breast cancer (BC) (*N* = 70).

	**Frequency**	**Percentage**
Age		
Below 50 years old	32	45.7
Above 50 years old	38	54.3
Marital status		
Married	46	65.7
Single	11	15.7
Divorced	4	5.7
Widowed	9	12.9
Education level		
Lower than secondary school	36	51.4
Higher than secondary school	34	48.6
Work		
Employed	19	27.1
Unemployed	51	72.9

### Measures and Ratings

The data were collected in hospitals in the summer of 2018, while patients were under treatment. The demographic information, such as age, educational background, marital status, work status, and insurance, were collected. Data collection instruments were all audio-taped under the consent of participants. DM was measured using the Defense Mechanism Rating Scale (DMRS) (Perry, 1990, 2000, 2014; Perry and Henry, 2004). The fourth level of the DMRS (psychotic) was not measured due to the nature of the sample and the inclusion criteria.

All procedures were performed in accordance with the 1964 Helsinki Declaration and its later amendments. Verbal informed consent was obtained from all participants who were involved in this study. This study was approved by the Ethical Committees from Northeast Normal University (NENU, China) and the Public Relations and Health Education Department in the Ministry of Public Health in Lebanon. Administrative approvals were taken from the ethical board, research center, or head of nurses in each of the six hospitals (NENU reference number: 2017003, MOPH official approval is present upon request, USJ reference number: CEHDF 1209, and verbal approvals were taken from other hospitals).

#### Defense Mechanism Rating Scale

The DMRS is a hierarchal DM rating scale that is based on the three levels of DMs or tripartite categories as follows: high adaptive or mature (level 7), neurotic (levels 5 and 6), and immature (levels 1–4). These three tripartite categories are separated into seven levels, which contain 30 defenses, and lower adaptive levels are related to higher anxiety and psychological impairment, while higher adaptive defenses are related to less anxiety and few psychological impairments. The Relationship Anecdote Paradigm (RAP) interview was used (Luborsky and Crits-Christoph, 1990). The semi-structured open-ended interviews of three spontaneous recent life vignettes included initial diagnosis, family, and daily life. Since the RAP interviews were conducted in Arabic, the first author translated the RAP interviews from Arabic into English. The English version was then cross-checked by translating it from English back to Arabic by a psychologist who is bilingual in both languages. Consequently, through the RAP, DMs of patients are defined into three-level scores presented in Tables 2, 3. Individual defense score is the number of times each defense is used. Defense level score allows comparison within the sample. The ODF is the reflection of the level of maturation of each participant (Perry, 2000). Two independent raters evaluated the RAP interviews, the first author, and a psychologist. The two raters then reached a consensus on which DMs are displayed by each participant. A good degree of reliability was found between DM group ratings. The average measure of interclass correlation coefficient (ICC) was 0.778 with a 95% CI from 0.636 to 0.865, *F*_(64, 64)_ = 4.508, and *p* < 0.001.

**Table 2:** Prevalence of defense levels between women patients with stages 2 and 4 BC.

	**Frequency of any use**				**Mean percentage use**			
	**Stage 2 (** ***n*** **=** **31)**		**Stage 4 (** ***n*** **=** **39)**		**Stage 2** **(** ***n*** **=** **31)**		**Stage 4** **(** ***n*** **=** **39)**	
**Defense levels**	**n**	**%**	**n**	**%**	**n**	**%**	**n**	**%**
7. High adaptive	31	100.0	39	100.0	56.4^*^	11.1	48.2	7.1
6. Obsessional	22	70.9	24	61.5	6.6	1.3	7.0	1.0
5a. Hysterical	7	22.5	9	23.1	1.7	0.3	1.5	0.2
5b. Other neurotic	9	29	19	48.7	2.0	0.4	3.5	0.5
4. Minor image distorting	22	70.9	33	84.6	8.8^*^	1.7	13.4	1.9
3. Disavowal	29	93.5	30	76.9	16.7	3.3	20.1	2.9
2. Major image distorting	17	54.8	18	46.1	4.3	0.8	3.4	0.5
1. Action	11	35.5	13	33.3	3.8	0.7	2.8	0.4
Tripartite categories								
High adaptive (level 7)	31	100.0	39	100.0	56.4^*^	11.1	48.2	7.1
Neurotic (levels 5–6)	24	77.4	31	79.5	10.3	2.0	12.0	1.8
Immature (levels 1–4)	31	100.0	39	100.0	33.6	6.6	39.7	5.9
Summary scores								
No. of defenses identified					29	5.7	30	4.45
Overall defense functioning					5.5	1.1	5.25	0.8

*p value for mean scores is by Wilcoxon rank-sum test.*

* *p < 0.05*.

**Table 3:** Prevalence of individual defenses between women patients with stages 2 and 4 BC.

	**Frequency of any use**				**Proportional scores**			
	**Stage 2** **(** ***n*** **=** **31)**		**Stage 4** **(** ***n*** **=** **39)**		**Stage 2** **(*n* = 31)**		**Stage 4** **(*n* = 39)**	
**Defense levels**	**n**	**%**	**n**	**%**	**M%**	**SD**	**M%**	**SD**
(7) High adaptive								
Affiliation	26	83.9	33	84.6	12.5	11.2	11.0	7.1
Altruism	8	25.8	14	35.9	1.8	3.4	3.1	4.4
Anticipation	16	51.6	15	38.5	4.5	5.9	2.6	3.7
Humor	8	25.8	12	30.8	3.6	6.8	2.8	5.3
Self-assertion	20	64.5	30	76.9	5.9	5.2	7.5	6.4
Self-observation	26^*^	83.9	24	61.5	9.3^**^	6.6	6.5	7.1
Sublimation	28	90.3	29	74.4	11.8	7.9	9.3	7.7
Suppression	18	58.1	25	64.1	7.0	7.2	5.4	5.8
(6) Obsessional								
Isolation	5	16.1	9	23.1	0.9	2.2	1.7	3.3
Intellectualization	14	45.2	18	46.2	3.9	5.5	4.9	6.2
Undoing	7	22.6	3	7.7	1.8^**^	3.5	0.37	1.3
(5a) Hysterical								
Repression	7	22.6	7	17.9	1.7	3.4	1.1	2.6
Dissociation	0	0.0	3	7.7	0.0	0.0	0.5	1.7
(5b) other neurotic								
Reaction formation	4	12.9	5	12.8	0.6	1.7	0.9	2.6
Displacement	7	22.6	14	35.9	1.4	2.7	2.5	4.0
(4) Minor image distortion								
Devaluation of self	9	29	15	38.5	2.0	3.6	2.9	4.4
Devaluation of others	19	61.3	28	71.8	5.3^*^	6.1	8.4	6.4
Idealization of self	2^*^	6.5	10	25.6	0.6	1.9	1.5	2.9
Idealization of others	4	12.9	4	10.3	0.5	1.4	0.5	1.5
Omnipotence	2	6.5	1	2.6	0.3	1.1	0.1	0.5
(3) Disavowal								
Denial	3	9.7	8	20.5	1.0	2.8	1.8	4.2
Projection	9	29	10	25.6	2.9	4.9	1.9	3.6
Rationalization	28	90.3	37	94.9	12.5	8.5	15.3	7.6
Autistic fantasy	3	9.7	6	15.4	0.7	2.4	1.1	2.8
(2) Major image distortion								
Splitting of others image	9	29	14	35.9	2.2	3.6	2.1	3.5
Splitting of self image	9	29	7	17.9	1.9	3.2	1.3	3.1
Projective identification	1	3.2	1	2.6	0.1	0.7	0.1	0.5
(1) Action								
Help-rejection	8	25.8	9	23.1	2.2	4.1	1.4	2.9
Acting out	4	12.9	3	7.7	0.9	2.6	0.9	4.7
Passive aggressive	3	9.7	2	5.1	0.7	2.3	0.5	2.3

*p value for frequency table is determined by using the Pearson's chi square test, while the p value for mean scores is determined by using the Wilcoxon rank-sum test.*

* *p < 0.05,*

** *p < 0.06*.

### Data Analysis

All data were analyzed using IBM SPSS version 22 software. Inter-rater reliability was calculated using the ICC. For comparison between groups, the repeated measures ANOVA and the Wilcoxon rank-sum test were used. The Spearman's rank was used for correlations. Finally, the effect size was measured using Cohen's *d* (Cohen, 1992).

## Results

There was difference in age, work, and marital status in terms of ODF and defense level scores. Participants below 50 years old (*M* = 5.5370, SD = 0.48605, *n* = 32) had significantly higher ODF (*z* = −2.195, *df* = 68, *p* = 0.028) than participants above 50 years old (*M* = 5.5370, SD = 0.48605, *n* = 38). The magnitude of difference in the means (mean difference: 0.312, 95% CI: 0.04291 to 0.58221) was medium (ɳ^2^ = 0.55). Moreover, participants below 50 years old (*M* = 5.9375, *SD* = 3.627) used significantly fewer immature defenses (*z* = −2.435, *df* = 68, *p* = 0.015) than participants above 50 years old (*M* = 6.973, SD = 3.483). The magnitude of difference in the means (mean difference: 8.43, 95% CI: −15.01074 to −1.67347) was medium (ɳ^2^ = 0.6). In terms of work, participants with BC who work (*M* = 2.237, *SD* = 3.521) used significantly more undoing (*z* = −2.158, *df* = 68, *p* = 0.012) than participants with BC who do not work (*M* = 0.549, SD = 2.023). The magnitude of difference in the means (mean difference: 1.688, 95% CI: 0.343 to 3.033) was medium to large (ɳ^2^ = 0.7). Furthermore, in terms of marital status, married patients with BC used significantly more mature defenses than unmarried patients with BC who did in five defense level scores. Results are presented in Table 4. In contrast, participants with a secondary or higher education did not show any significant difference in the DM used compared with participants with lower than secondary education. Also, there were no significant interactions between stages and age, *F*_(1, 1)_ = 1.086, *p* = 0.301, between stages and marital status, *F*_(1, 1)_, *p* = 0.494, between stages and work, *F*_(1, 1)_ = 0.723, *p* = 0.398, and between stages and educational level, *F*_(1, 1)_ = 0.966, *p* = 0.329.

**Table 4:** Differences in defense mechanism between married and single patients with BC.

	**Married**		**Unmarried**		***z***	***df***	***p***	**95% CI**		**ɳ^**2**^**
	**M**	**SD**	**M**	**SD**				**Lower**	**Higher**	
Altruism	3.467	4.505	0.708	1.944	−2.7	68	0.007	−4.686	−0.832	0.7
Self-observation	8.869	6.541	5.479	7.348	−2.325	68	0.02	−6.819	0.039	0.5
Idealization of self	1.5	2.912	0.5	1.225	2.073	68	0.038	−2.493	−0.007	0.5
Denial	1	3.347	2.375	4.011	−2.022	68	0.043	−0.426	3.176	0.4
Disavowal	16.706	9.086	22.208	8.785	−2.137	68	0.033	0.987	10.016	0.6

### Psychological Defenses

For patients with stage 2 BC, the percentage of using each defense level at least once fell into three ordinal groups as follows: high-adaptive and disavowal levels (100%−93.5%), obsessional, minor image distorting, and major image distorting levels (70.9%−54.8%), and the lowest action, hysterical, and other neurotic levels (35.5%−22.5%). For patients with stage 4 BC, the percentage distribution was slightly different as follows: high-adaptive and minor image distortion levels (100%−84.6%) were the highest, and disavowal, obsessional, other neurotic, and major image distortion levels (76.9%−46.1%), and action and hysterical levels (33.3%−23.1%) were the lowest. This is reflected at the tripartite levels for both groups. Moreover, this indicated that participants in both groups used each level of defense at least once. Individual defense and defense level are shown in Tables 2, 3, respectively. In both tables, the left columns represent the frequency and percentage of each DM used at least once in patients with stages 2 and 4 BC. The right columns represent the mean proportional scores in each of the two groups. Table 2 also represents the number of defenses identified in each group and the ODF scores.

The distribution of the mean proportional defense level scores was the same for both groups. Table 2 shows that patients with stages 2 and 4 BC used high-adaptive (56.4% and 48.2%, respectively) and disavowal levels (16.7% and 20.1%, respectively) the most, followed by minor image distortion (8.8% and 13.4%, respectively) and obsessional levels (6.6% and 7%, respectively), and major image distortion levels (4.3% and 3.4%, respectively), action levels (3.8% and 2.8%, respectively), other neurotic levels (2% and 3.5%, respectively), and hysterical levels (1.7% and 1.5%, respectively) the least. The mean proportion scores were similar at the tripartite level: high-adaptive and immature levels were the highest, while the neurotic level was the lowest. There were two significant differences between patients with stages 2 and 4 BC at the defense level scores. Patients with stage 2 BC used significantly more high-adaptive defense levels than patients with stage 4 BC, *z* = −2.129, *df* = 68, *p* = 0.033. The magnitude of difference in the means (mean difference: 8.245, 95% CI: 1.313 to 15.177) was medium (ɳ^2^ = 0.6). Conversely, patients with stage 4 BC used significantly more minor image distortion levels than patients with stage 2 BC (*z* = −2.241, *df* = 68, *p* = 0.25). The magnitude of difference in the means (mean difference: −4.575, 95% CI: −8.535 to −0.6154) was medium (ɳ^2^ = 0.55).

Table 3 represents individual defenses. The majority of both groups used five individual defenses at least once. Patients with stage 2 BC used sublimation, rationalization, affiliation, self-observation, and self-assertion in descending order of magnitude. The majority of patients with stage 4 BC used rationalization, affiliation, self-assertion, sublimation, and devaluation of others at least once in descending order of magnitude. There was a significant difference in the two individual defenses. Patients with stage 2 BC had higher percentages of self-observation, while patients with stage 4 BC had higher percentages of idealization of self (see Table 3 for details).

Furthermore, in Table 3, it is shown that patients with stage 2 BC had seven individual defenses with a display of more than 5% of the total defense functioning, the cutoff of frequent used. The highest means were affiliation and rationalization (12.5% each), then followed by sublimation, self-observation, suppression, self-assertion, and devaluation of others. Patients with stage 4 BC also had seven individual defenses with a display of more than 5% of the total defense functioning. The highest mean was rationalization (15.3%), then followed by affiliation, sublimation, devaluation of others, self-assertion, self-observation, and suppression. There were significant differences at the three individual defenses. Patients with stage 2 BC used more self-observation and intellectualization individual defenses, while patients with stage 4 BC used more devaluation of others (see Table 3 for details).

## Discussion

This study investigated the unconscious reaction and coping with the disease progress and process of patients with BC by measuring the difference in patients with stages 2 and 4 BC.

The results showed that DM differed in relationship with the social status. Younger patients with BC used more mature or high adaptive defenses and less immature defenses than older patients with BC. Thus, age played a role in using more mature or adaptive defenses. One probability is that younger patients with BC have a wider social surrounding and are more capable of movement or social interaction than older patients with BC. Our results align with previous studies as age was associated with self-efficacy to cope with BC (Collie et al., 2005). Similarly, married patients with BC used more altruism, self-observation, and idealization of self than unmarried patients with BC. Married patients with BC thus reacted toward BC by devoting themselves to their families and children, which helped them first by coping with BC and second by using a high adaptive defense instead of a low adaptive one. In contrast, unmarried patients with BC used more disavowal, rationalization being the highest percentage, and denial than married patients with BC.

### Psychological Defenses

The results showed similarities and differences in the level of DMs between patients with stage 2 and stage 4 BC.

There was a similarity in the order and magnitude of means of defenses used by both groups. All participants used high adaptive and immature levels at least once, while the majority of them used neurotic levels at least once. However, at the seventh hierarchal level, participants with stage 2 BC used at least one disavowal level than participants with stage 4 BC who used more major image distortion defense levels. In contrast, both groups used action and hysterical levels the lowest. Moreover, the mean proportion of the defense level scores showed that both groups used high adaptive and disavowal defense levels the most, while they used action and neurotic defense levels the least. Both groups, i.e., patients with stage 2 and stage 4 BC, displayed more or less similar mean magnitudes and distribution of defenses used at least once.

These similarities in defense mechanism display and defenses used at least once were also reflected in the individual DMs. The majority of participants in both groups used most of the individual defenses at least once. In addition, the majority of participants used high-adaptive defenses on a frequent basis, such as affiliation, sublimation, self-observation, and suppression. On the one hand, only rationalization and devaluation of others in the lower individual defenses were used on a frequent basis. These results imply that, in general, patients with BC reacted and dealt with the disease by mostly using high-adaptive levels of DMs. Unconscious coping with BC was by using affiliation to the family, religious sublimation, suppression of cancer toward a higher cause: family in this case, and self-observation. On the other hand, the use of the lower individual defenses as rationalization of the disease helped patients with BC in coping with the disease, similarly to devaluation of others.

Alternatively, there were five differences between patients with stage 2 and stage 4 BC, which contradicted previous studies that did not find differences among BC stages (Beresford et al., 2006; Perry et al., 2015). Patients with stage 2 BC used more defenses in high adaptive levels than patients with stage 4 BC. At the individual defense level, patients with stage 2 BC relied more on self-observation and undoing than patients with stage 4 BC. Both self-observation and undoing are considered at the top of the maturity or adaption levels of defense functioning. Conversely, patients with stage 4 BC rely more on idealization of self and devaluation of others than patients with stage 2 BC at the lower adaptation level. Therefore, there is a difference in adaptation level with BC between patients with stage 2 and stage 4 BC, where patients with stage 2 BC relied more on mature individual defenses. This is reflected at the seventh hierarchal level and tripartite level, where patients with stage 2 BC used more defenses in high adaptive levels while patients with stage 4 BC used more defenses in minor image distorting levels. Thus, there seems to be a relationship between the stages of BC and the use of defenses in high adaptive levels. Further research might have supplementary results on the difference among different BC stages, which might give us a better understanding of these differences or how different stages of patients with BC unconsciously cope with the disease.

### Conclusion

In general, although the distribution of defenses used at least once and the mean magnitude of defenses is similar between stages 2 and 4, patients with more developed cancer stage used lower adaptive DMs. Moreover, younger patients tend to adapt to BC by using higher adaptive defenses than older patients. A similar conclusion is found in social status. For example, when being in a responsible situation as in having a family to take care of, patients with BC tend to use higher adaptive defenses than those with lesser responsibilities. Hence, the severity of BC, the age of carriers, and the social status may lead to higher use of adaptive DMs at the individual defense and the hierarchal or the tripartite levels. Moreover, the overall unconscious coping with BC was mostly through high-adaptive mechanisms. This element can be focused on therapies or counseling of patients with BC through their disease progress.

### Limitations of This Study

This study has three main limitations. The first limitation is the sample size, and a bigger sample can be more representative, although Lebanon is of a small population. The second limitation is that having a longitudinal study would have enriched the results and allowed us to see the development of DMs, mainly because defenses change and develop over time. The third limitation is that the research did not compare patients with BC with a sample of patients with non-BC. However, this is due to the absence of national data and statistics that will allow us to gather a comparable sample.

### Implications for Psychosocial Providers or Policy

Having a general overview of psychosocial factors displayed in a social context allows us to form the base of assisting in BC or cancers in general. The assistance is limited not only to the medical sector, such as medical professionals, but also to the wider social interactions and personals involved or affected throughout the process. Thus, based on this study, further research of implication can give various applicable processes of psychological treatment in cases of cancers in general or BC in specific.

## Data Availability Statement

The datasets used and/or analyzed during the current study are available from the corresponding author on reasonable request.

## Ethics Statement

The studies involving human participants were approved by the ethical committees from Northeast Normal University (NENU126 China) and the Public Relations and Health Education Department in the Ministry of Public Health. The participants provided their written informed consent to participate in the study.

## Author Contributions

MS and XH contributed conception and design of the study. MS collected the data. MS and MH contributed to the procedure and rating of the study. MS performed the statistical analysis. MS and XH contributed to manuscript revision, read, and approved the submitted version. All authors have read and approved the final manuscript.

## Conflict of Interest

The authors declare that the research was conducted in the absence of any commercial or financial relationships that could be construed as a potential conflict of interest.

## Publisher's Note

All claims expressed in this article are solely those of the authors and do not necessarily represent those of their affiliated organizations, or those of the publisher, the editors and the reviewers. Any product that may be evaluated in this article, or claim that may be made by its manufacturer, is not guaranteed or endorsed by the publisher.
